# The Impact of Antidiabetic Agents on Sarcopenia in Type 2 Diabetes: A Literature Review

**DOI:** 10.1155/2020/9368583

**Published:** 2020-07-09

**Authors:** Chen-Ning Wu, Kai-Jen Tien

**Affiliations:** ^1^Department of Internal Medicine, Chi Mei Medical Center, Tainan, Taiwan; ^2^Division of Endocrinology and Metabolism, Department of Internal Medicine, Chi Mei Medical Center, Tainan, Taiwan

## Abstract

Sarcopenia is a geriatric syndrome characterized by decline of skeletal muscle mass and function. Contributing factors include nutritional, genetic, inflammatory, and endocrinal factors. The reported prevalence of sarcopenia in type 2 diabetes mellitus is high, especially in patients with poor glycemic control. Additionally, antidiabetic agents may alter the balance between protein synthesis and degradation through various mechanisms of skeletal muscle mass regulation. This study reviewed the literature on the pathogenesis of sarcopenia in diabetes mellitus and the current understanding of whether antidiabetic agents contribute positively or negatively to sarcopenia and muscle wasting.

## 1. Introduction

Sarcopenia, the age-associated loss of skeletal muscle mass and function, is a common condition in the elderly. Adverse health outcomes associated with sarcopenia include physical disability, poor quality of life, and mortality. A recent study also reported that obese patients with sarcopenia have a 2.63-fold higher risk of cardiovascular disease compared to a control group of obese patients without sarcopenia [1]. Thus, healthcare professionals must be able to recognize and prevent the development of sarcopenia when treating elderly and/or obese patients. Although universally accepted diagnostic criteria for sarcopenia have not been established, a sarcopenia diagnosis is usually based on assessments of muscle mass, muscle strength, and physical performance. Appendicular muscle mass, grip strength, and gait speed are among the most useful measurement tools. Multiple factors can contribute to development of sarcopenia, including nutritional deficiency, genetics, inflammation, and endocrinal factors [2]. Advanced age, low body mass index, insufficient physical activity, and chronic diseases are also known risk factors for sarcopenia [3].

## 2. Sarcopenia Pathophysiology

The pathophysiology of sarcopenia involves an imbalance between anabolic and catabolic pathways that regulate skeletal muscle mass [2]. Sarcopenia occurs when the rate of muscle protein degradation exceeds the rate of muscle protein synthesis.

The major anabolic pathway involves activation of the Akt mammalian target of rapamycin (mTOR), which increases muscle protein synthesis [4]. In the fed state, this anabolic pathway can be triggered by high levels of circulating amino acids, which activate mTOR via RAG GTPases [5]. Other factors that provoke upregulation of this pathway are factors that promote muscle growth, e.g., insulin-like growth factor 1 (IGF-1), testosterone, *β*2-adrenergic agents, and exercise [6].

The major catabolic pathways participate in activation of ubiquitin proteasome, calpain, or caspase pathways, which are under transcriptional control of the transcription factors forkhead box O and nuclear factor- (NF-) *κ*B [7]. In patients with obesity, immobility, inflammation, cancer, or other chronic diseases (e.g., heart failure, chronic obstructive pulmonary disease, and chronic kidney disease), transcription factor NF-*κ*B is activated by increases in proinflammatory cytokines (e.g., interleukin-6, interleukin-1, and/or tumor necrosis factor alpha) [8]. Some chronic diseases are known to impair muscle regeneration by degrading mitochondrial function and by exhausting the repair capacity of resident satellite cells. Age-associated declines in anabolic hormones (e.g., testosterone, growth hormone, and IGF-1) can inhibit Akt-mTOR and decrease protein synthesis [9]. Multiple factors can cause sarcopenia in elderly patients, particularly mitochondrial dysfunction, oxidative stress, a proinflammatory state, and metabolic inefficiency. Malnutrition resulting in protein and glucose levels insufficient for muscle synthesis is another possible cause of sarcopenia.

## 3. Diabetes Mellitus and Sarcopenia

A high prevalence of sarcopenia has been reported in elderly patients with type 2 diabetes mellitus (T2DM). In Japan, one out of six elderly patients with T2DM has sarcopenia [10]. In Korea, Kim et al. reported that patients with T2DM had a 3-fold higher risk of sarcopenia compared to those without T2DM [11]. Compared to western populations, Asian populations are more prone to abdominal obesity, low muscle mass, and increased insulin resistance, all of which are known risk factors for diabetic sarcopenia. Therefore, physicians in Asia must be proactive in identifying and mitigating or preventing the emergence of these risk factors [12]. In Japan, a study of diabetic patients reported that poor glycemic control was associated with low skeletal muscle mass [13]. Patients who have both low muscle mass and T2DM are two times more likely to perform poorly on physical performance tests (e.g., gait speed test, grip strength test, and Timed Up and Go test) compared to patients who have neither of these factors [14]. In older adults with T2DM, glucose fluctuations are reportedly associated with sarcopenia after adjustment for HbA1c level [15]. Dysglycemia contributes to loss of skeletal muscle mass, loss of strength, and decreases in overall physical performance through four mechanisms. First, insulin plays a pivotal role in muscle functioning by increasing glucose uptake and by promoting intracellular glucose metabolism [16]. Thus, insulin resistance can impair muscle strength. Second, insulin resistance reportedly downregulates the mTOR pathway. The resulting decrease in protein synthesis then reduces the protein available for muscle anabolism. Third, cytokines associated with chronic inflammation contribute to insulin resistance, lipolysis, muscle protein breakdown, and nitrogen loss in T2DM [17]. Fourth, chronic hyperglycemia, which increases accumulation of advanced glycation end-products, contributes to reduced muscle strength [18].

## 4. Antidiabetic Agents in Sarcopenia

### 4.1. Metformin

Activation of AMP-activated protein kinase (AMPK) by metformin inhibits the mTOR complex, which reduces anabolic effects. Metformin causes the activation of the forkhead box protein O axis through AMPK activation and induces autophagy and protein degradation [19]. However, metformin improves insulin sensitivity and potentiates insulin actions in the skeletal muscle, which may be mediated by upregulation of peroxisome proliferator-activated receptor-*γ* coactivator 1-*α* [20]. Metformin also reduces muscle lipid accumulation and stimulates mitochondrial biogenesis, angiogenesis, and preservation of oxidative muscle fibers [21]. Inhibition of NF-*κ*B signaling by AMPK activation suppresses the inflammatory response, which may depress muscle protein breakdown [19]. Activation of AMPK has revealed a multitude of health benefits in recent studies of sarcopenia [22]. A double-blind randomized controlled trial disclosed that metformin increased walking speed in nondiabetic and nonfrail elderly patients, which implies that metformin positively affects lower limb strength [23]. In a prospective cohort study of elderly women with diabetes in the United States, declines in walking speed were lower in women who had taken metformin compared to controls [24]. Another cohort study disclosed that an insulin sensitizer such as metformin may attenuate lean body mass loss during the aging process in older men with diabetes [21]. Although its precise mechanisms are not clearly understood, metformin apparently has positive effects on both muscle mass and muscle strength. Further well-designed studies are needed to elucidate the effects of metformin in sarcopenia.

### 4.2. Sulfonylurea

The primary mechanism of sulfonylurea is closure of the ATP-sensitive K-channel (K_ATP_ channel) in the beta-cell plasma membrane, which initiates a chain of events resulting in insulin release. The K_ATP_ channel activity in response to tissue stress maintains cell integrity and homeostasis. The roles of K_ATP_ channel openers have been studied in a diverse spectrum of therapeutic applications, including myopreservation, vasodilatation, cytoprotection, and islet cell protection [25]. Closure of the K_ATP_ channel via sulfonylurea induces *β* cell apoptosis in human islets and may decrease *β* cell mass. Although the exact mechanism of this phenomenon is uncertain, closure of K_ATP_ and Ca^2+^-dependent exocytosis may translate into apoptotic signals in cells [26]. Tricarico et al. demonstrated that incubation with the K_ATP_ channel blocker glibenclamide may activate atrophic signaling in rat skeletal muscles and that these effects can be prevented by diazoxide, a K_ATP_ channel opener [27]. Blockade of the K_ATP_ channel activates atrophy in skeletal muscles through caspase-3-dependent or independent pathways [27]. Furthermore, the combined activities of glibenclamide and other cytotoxic drugs with atrophic effects may be associated with a toxicodynamic drug-drug interaction in muscle tissue [27]. An in vivo study suggested that hypoglycemia may also cause neuron death and skeletal muscle damage through various pathways [28, 29].

One “in vitro” study of sulfonylureas and glinide-induced atrophy in skeletal muscles elucidated their efficacy in reducing the protein content in the flexor digitorum brevis (FDB) muscle in mice. The study revealed that the relative efficacy of diabetes medications in reducing the protein content in the FDB muscle after 24 hours of incubation time is as follows: repaglinide ≥ glibenclamide > glimepiride > tolbutamide > nateglinide [30]. A search of atrophy-related signaling in the Food and Drug Administration Adverse Event Reporting System (FDA-AERS) showed that muscle atrophy occurred in 0.27% of human subjects who had used glibenclamide, whereas no atrophy was reported in subjects who had used sulfonylureas or glinides [30].

One unanswered question is why compounds (e.g., atrophic agents and KATP channel blockers) that have revealed mechanisms similar to those of SU in “in vitro” studies do not reveal similar adverse effects in human studies. One possibility is that their clinical manifestations may be too subtle to detect in human studies. Additionally, even if adverse effects are observed, the researcher may not consider them important enough to mention in the FDA-AERS report. Given the limited evidence provided by human studies, we cannot conclude that SU and glinides are atrophic agents. However, the “in vitro” data indicate that SU, particularly glibenclamide and glinides, should be used with extreme caution to minimize sarcopenia risk in vulnerable patients.

### 4.3. Thiazolidinedione

Insulin resistance reportedly accelerates muscle protein degradation. Thiazolidinedione, a synthetic ligand for peroxisome proliferator-activated receptors (PPARs), improves glycemic control by reducing insulin resistance in the liver and peripheral tissues, which is believed to attenuate muscle protein degradation. A db/db mouse study of rosiglitazone concluded that it increases circulating adiponectin, improves insulin resistance, corrects abnormal PI3K/Akt signaling, and reduces caspase-3 pathways leading to suppression of proteolysis and atrophy [31, 32]. In the skeletal muscle, pioglitazone improves energy metabolism by reducing intramyocellular lipid content and by improving fatty acid metabolism [33]. In women with polycystic ovary syndrome, pioglitazone also enhances mitochondrial biogenesis and ribosomal protein biogenesis in the skeletal muscle [34]. Although thiazolidinedione should theoretically have beneficial effects on skeletal muscle physiology in insulin-resistant subjects, clinical data for the effects of thiazolidinedione on sarcopenia-related parameters are scarce and controversial. A study of weight loss in nondiabetic overweight/obese adults revealed that pioglitazone increased visceral fat loss but had no effect on skeletal muscle loss. Resistance training, however, significantly decreased skeletal muscle loss in the study population [35]. Interestingly, pioglitazone potentiates the effect of resistance training on muscle strength in women but not in men [36]. In another study, diabetic men given pioglitazone alone did not significantly differ from normoglycemic men in terms of total lean mass loss or appendicular lean mass loss over a mean 3.5 years of follow-up [21]. It remains unclear why the clinical outcomes in our study were not compatible with current biochemical theory, and further well-designed studies are warranted.

### 4.4. Sodium/Glucose Cotransporter 2 Inhibitor

Sodium/glucose cotransporter 2 inhibitor (SGLT2i) represents a novel class of glucose-lowering agents that prevents reabsorption of glucose from the kidney and facilitates its excretion in urine, independent of insulin secretion and action. Notably, SGLT2i is associated with some adverse events, including urogenital infection, dehydration, and ketosis/ketoacidosis. However, due to its overall beneficial effects on cardiovascular and renal outcomes in randomized-controlled trials, SGLT2i has been recommended for use in treating T2DM patients with heart failure and/or chronic kidney disease [37].

Another concern is that SGLT2i may induce diabetes-associated sarcopenia. Yabe et al. reported that decreased serum insulin and increased glucagon levels caused by SGLT2i reduce muscle uptake of glucose and amino acids and enhance proteinolysis. Chronic SGLT2i use may also accelerate diabetes-associated sarcopenia [38]. Yasuda et al. reported a case of a lean elderly patient diagnosed with sarcopenia after taking dapagliflozin for 1 year to treat T2DM [39]. Another study reported that a 6-month treatment with ipragliflozin significantly reduced arm muscle mass but not total muscle mass [40]. Similarly, Tsurutani et al. demonstrated a clinically significant decrease in skeletal muscle mass index in an ipragliflozin group compared with a sitagliptin group [41]. Another case report disclosed that empagliflozin treatment induced myopathy in patients and that the myopathy improved when the patients stopped taking the medication [42]. Clinical data are currently too limited to determine how frequently SGLT2i-related sarcopenia occurs and whether SGLT2i causes diabetes-associated sarcopenia.

Different studies have reported different effects of SGLT2i. For example, one study disclosed that lean body mass did not decrease in diabetic db/db mice given SGLT2i. The authors hypothesized that long-term use of SGLT2i may rescue energy storage in muscle tissue by improving insulin sensitivity and by preserving endogenous insulin secretions that counteract muscle catabolism [43]. Sano et al. reported increased hand grip strength in both men and women treated with SGLT2i for 10 weeks [44]. In another study, an experimental group treated with dapagliflozin had significantly larger reductions in body fat mass over a 102-week period in comparison with a control group treated with placebo (lean tissue mass did not significantly differ between groups) [45]. Another original article reported that a 6-month dapagliflozin treatment in Japanese patients with T2DM significantly improved glycemic control and reduced body weight and fat mass without reducing skeletal muscle mass [46]. Canagliflozin reduced fat mass and hepatic fat content without significantly reducing muscle mass in another study of a Japan population of T2DM patients with nonalcoholic fatty liver disease [47]. An observational study similarly reported that SGLT2i (empagliflozin or dapagliflozin) reduces adipose tissue mass without affecting lean tissue parameters [48].

Current theories indicate that mTOR inhibition during night time fasted state enhances autophagy of dysfunctional organelles and improves mitochondrial functions that support the metabolic switch. In the daytime fed state, mTOR is activated by high levels of circulating amino acids, which leads to an anabolic state characterized by autophagy inhibition, protein synthesis, and mitochondrial biogenesis. Healthy patients have a balanced cycle of anabolism and catabolism. In contrast, T2DM patients on insulin therapy experience increased insulin levels that lock the patients in an anabolic state and prevent glucagon increases, which reduces its beneficial catabolic effects. Use of SGLT2i restores overnight glucagon release and catabolic benefits while allowing restoration of the anabolic state during daytime, which reinstates the diurnal metabolic cycle and improves metabolic health [5]. Further human studies are needed to investigate the effects of these drugs on sarcopenia.

### 4.5. Incretin-Based Antidiabetic Agent

In a mouse study, anagliptin improved skeletal muscle glucose uptake by enhancing insulin-induced capillary recruitment and interstitial insulin concentrations via an NO-dependent mechanism [49]. Another animal study reported similar results for glucagon-like peptide 1 (GLP-1) [50]. Dipeptidyl peptidase-4 (DPP4) inhibitors upregulate glucose transporter type 4 (GLUT4) in the heart and skeletal muscle in spontaneously hypertensive rats [51]. Upregulation of GLUT4 improves skeletal muscle glucose uptake by diffusing circulating glucose into muscle and fat cells. The underlying mechanism of sitagliptin-induced upregulation of GLUT4 may be partially attributable to GLP-1 [51]. Rajaobelina et al. reported that DPP4 inhibitors attenuate the decline in skeletal muscle mass in T2DM [52]. Sarcopenic parameters (fat-free mass, skeletal muscle mass, and related indices, muscle strength, and gait speed) in elderly diabetic patients are superior in patients treated with DPP4 inhibitors for at least 24 months compared to those treated with SU for the same period [53]. Liraglutide effectively induces loss of fat and increased skeletal muscle index in elderly T2DM patients who are overweight or obese [54].

However, one study reported significant loss of fat mass and skeletal muscle mass in T2DM patients on hemodialysis after 6 months of dulaglutide treatment [55]. Although most studies of liraglutide have disclosed positive effects on muscle mass, it is currently unknown whether the effects of DPP4 inhibitors on sarcopenia are through a direct or indirect (i.e., through GLP-1 increase) mechanism.

### 4.6. Insulin

Tanaka et al. disclosed that reduction in endogenous insulin secretion is a risk factor for sarcopenia in men with T2DM. The authors reported that all parameters for endogenous insulin had significant positive associations with muscular parameters [56]. Bouchi et al. performed a retrospective observational study of the impact of insulin treatment on muscle mass in T2DM. After propensity score matching, the insulin treatment group had a significantly higher skeletal muscle index compared to the control group [57]. Current clinical evidence indicates that insulin treatment could attenuate the deterioration of sarcopenia in T2DM patients.

## 5. Conclusions

Sarcopenia is emerging as an important healthcare issue in T2DM patients. Sarcopenia can have multiple causal factors, but factors that should raise a suspicion of sarcopenia in T2DM patients include old age, chronic conditions, functional decline, unintentional weight loss, depressive mood, cognitive impairment, repeated falls, and malnutrition [58]. Growing evidence suggests that antidiabetic agents have important roles in muscle physiology. This literature review indicates that antidiabetic agents may have some impacts on muscle mass and performance in T2DM. However, T2DM is a highly heterogeneous disease; it is difficult to determine which is a greater contributor to sarcopenia: T2DM per se or prescribed medications. That is, current evidence is insufficient to support recommendations or prohibitions for these patients. More research is needed to clarify the relationship between antidiabetic agents and sarcopenia.
